# Driving carbon emission reduction in China through green finance and green innovation: an endogenous growth perspective

**DOI:** 10.1007/s11356-024-32067-w

**Published:** 2024-01-26

**Authors:** Kunming Li, Weiyuan Lin, Tingjun Jiang, Yifan Mao, Wenming Shi

**Affiliations:** 1 College of Economics and Management, Fujian Agriculture and Forestry University, Fuzhou, Fujian 350002 People’s Republic of China; 2 Center for Maritime and Logistics Management, Australian Maritime College, University of Tasmania, Launceston, TAS 7250 Australia

**Keywords:** Green finance, Green innovation, Carbon emissions, Endogenous growth model, Moderating effect

## Abstract

Discovering drivers of carbon dioxide (CO_2_) emissions is vital for the Chinese government to achieve carbon peak and carbon neutral. With this aim, a theoretical endogenous growth model capturing the mitigating effect of green finance and green innovation on carbon emissions is constructed in this study, which is further empirically examined using China’s municipal-level panel data during 2010–2019. The main findings are as follows: First, there is theoretical and empirical evidence supporting that green finance and green innovation can inhibit carbon emissions. Second, the above inhibitory effects demonstrate clear regional disparities with significant effects only in eastern and central Chinese cities, which are moderated by environmental regulations and marketization levels, respectively. Third, in cities with high green finance, green finance plays a more significant role in reducing carbon emissions than green innovation, and the opposite is true in cities with low green finance. In addition, the robustness and endogeneity checks indicate that the results of this study are robust and reliable. These theoretical and empirical findings create profound implications for CO_2_ emission reduction by vigorously guiding funds to green finance and formulating scientific and effective environmental regulations to promote green innovation in China.

## Introduction

In recent years, the world has faced more and more prominent environmental problems caused by global warming with excessive carbon dioxide (CO_2_) emissions and other greenhouse gases being the main cause. Such changes in global climate have become a worldwide concern and call for global collaboration for CO_2_ emission reduction (Montzka et al. 2011). As one of the largest CO_2_ emitting countries, China’s economy is currently shifting from “high growth” to “high quality development” and has been experiencing ever-increasing challenge of CO_2_ emission reduction. To cope with this challenge, the Chinese government has made great efforts and significantly reduced CO_2_ emissions. However, due to the dominance of coal consumption and a low energy utilization efficiency, China’s total CO_2_ emissions remain high. Referring to the International Energy Agency (IEA; https://www.iea.org/), China’s carbon emissions in 2021 accounted for 32.78% of the world’s carbon emissions and reaching per capita carbon emissions of 8.4 t. At the United Nations Climate Change Conference in Glasgow (COP26) in 2021, the participating countries successfully signed the Glasgow Climate Pact, reaffirmed their emission reduction commitment to the Paris Agreement, and reached a consensus on CO_2_ emission reduction and green development. Notably, China has also clearly put forward its “double carbon” strategy, aiming to achieve 2030 carbon peak and 2060 carbon neutrality.

To implement the double carbon strategy, China’s development must rely primarily on the low-carbon mode. As the blood of economic development, it has been extensively believed that finance is vital to promote green economic development by providing large amounts of capital investment and continuous fiscal and financial policy support, which accelerates the emergence of green finance (Sadiq et al. 2022). The G20 Green Finance Group defined green finance as the investment and financing activities that can bring benefits to the environment and promote development in a sustainable manner (Ma et al. 2016). To promote green economic transformation, green finance provides enterprises with green financial services and stimulates green technology innovation by reducing their financing costs and offering financial policy support. At present, China has obtained fruitful achievements, including the establishment of a green financial framework with “three functions” and “five pillars” and the implementation of a series of green financial policies for supporting ecological environmental protection (Muganyi et al. 2021), addressing climate change (Zhang et al. 2022a, b), and enhancing efficient use of resources (Lee and Lee 2022). These achievements significantly improve the top-level design and development of green finance.

Despite the above policy-related achievements, some realistic questions are raised. For example, how much can green finance contribute to CO_2_ emission reduction? What is the moderating mechanism through which green finance inhibits CO_2_ emissions? To answer these questions, this study examines how green finance and green innovation drives carbon emission reduction in China from an endogenous growth perspective.

Consequently, this study can make important contributions to the existing literature from the following three perspectives: First, the construction of an endogenous growth model including green finance, green innovation, environmental regulations, and CO_2_ emissions contributes to the role of green finance and green innovation in inhibiting carbon emissions from a theoretical perspective. Second, using China’s municipal-level panel data during 2010–2019, the above inhibitory effects as well as the moderating mechanisms of environmental regulations and marketization levels are further empirically validated. This confirms that green finance and green innovation can be an efficient tool for reducing carbon emissions in China from an empirical perspective. Third, theoretical and empirical findings of this study provide rich policy implications for China to shift its development to the green and low-carbon mode through green finance, which serves as a reference to promote sustainable development in other countries.

The remainder of this study is as follows: “Literature review” section provides a thorough review of factors and mechanisms of carbon emissions. “Endogenous growth model and research hypotheses” section constructs a theoretical endogenous growth model capturing how green finance and green innovation drive carbon emission reduction and develops three hypotheses. “Methodology” section presents the variables, data sources, and the benchmark model for validating the proposed hypotheses. “Empirical results and discussions” section reports the results of the benchmark model, moderating mechanism analysis, robustness confirmation, and endogeneity check. “Conclusions” section concludes this study by summarizing the main findings, discussing policy implications, and pointing out future research directions.

## Literature review

The existing studies have thoroughly investigated the influential factors of CO_2_ emissions as well as the pathways to reduce carbon emissions and identified a range of drivers such as economic growth (Grossman and Krueger 1995), technological innovation (Ganda 2019), industrial structure, urbanization (Answer et al. 2020), and financial development (De Haas and Popov 2019). Likewise, to implement the double carbon strategy in China, tremendous efforts have been made to reduce carbon emissions, bringing fruitful achievements. For example, Zheng et al. (2019) confirmed the role of economic growth in affecting CO_2_ emissions in China and found that China’s slow growth rate of CO_2_ emissions in recent years is largely due to the improvement of energy efficiency and the transition to a consumption-driven economy. Meanwhile, carbon emissions usually increase as urbanization accelerates in China (Zhou et al. 2021), but they decrease as technological innovation enhances (Khan et al. 2020). Regarding how finance drives CO_2_ emission reduction, Shahbaz et al. (2013) identified the mechanisms of corporate technological innovation enhancement and environmental awareness improvement through which financial development reduces carbon emissions. Sachs et al. (2019) and Hafner et al. (2020) supported that green financial instruments significantly reduce carbon emissions through encouraging investments in low-carbon technologies and alternative renewable energies, which further protects the environment and promotes green development. However, Acheampong et al. (2020) and He et al. (2020) provided inconsistent findings. They supported that the relationship between financial development and CO_2_ emissions can be depicted as an inverted U-shaped curve.

Along with the vigorous promotion of green finance policies in China, previous studies have paid ever-rising attention to the inhibitory impact of green finance on CO_2_ emissions. Using a difference-in-difference model, Su and Lian (2018) focused on heavy-polluting enterprises and examined how green credit policy influenced their investment and financing behavior. They found that green credit can direct funds to industries with priorities of energy-saving and environmental protection. Jiang et al. (2020) supported that green credit and green venture capital can mitigate carbon emissions from the following two perspectives: One is to alleviate green enterprises’ financing constraints and provide them with more low-carbon products or services. The other is to reduce funds for enterprises with high pollution and high emissions. Zhang et al. (2022a, b) found that the industrial carbon emission intensity of heavily polluting enterprises decreased by 0.267 t/104 yuan per year on average after the implementation of green credit policy, and they identified the green innovation effect as a potential mechanism of the above impact. Similar findings on the role of green finance in reducing carbon emissions have also been obtained in other economies in the world. For example, Meo and Karim (2022) proved that green finance is a key factor of carbon emission reduction using evidence from 10 major economies such as the USA, the UK, and Canada. The necessity of developing green finance to reduce carbon emissions was also confirmed by Bakry et al. (2023) using the data on 76 developing countries and Umar and Safi (2023) using the data on the Organization for Economic Co-operation and Development (OECD) countries. Overall, it has been widely recognized that green finance can greatly contribute to CO_2_ emission reduction in both developing and developed economies.

As an important driver of energy saving and green transformation, green innovation has been extensively incorporated into the framework of green finance, aiming to reduce carbon emissions. As a result, previous studies pay considerable research attention to examine how green finance effectively promotes green innovation and obtain consistent findings. Li et al. (2018) theoretically proved that green credit can promote corporate green innovation. Based on a quasi-natural experiment regarding green finance reform and innovation pilot zone construction in China, Li and Liu (2021) confirmed a significantly positive effect of green finance policies on corporate green innovation. Similar findings are provided by Xie (2021) using evidence from Chinese A-share manufacturing-listed companies and Wang and Wang (2021) based on the difference-in-difference evidence from issuing “Green Credit Guidelines.” Cao et al. (2021) provided further evidence from a heterogeneity perspective, indicating that the inhibitory impact of green credit on heavy-polluting enterprises’ green innovation decreases with the improvement of their corporate social responsibility ratings. According to a study of 48 countries, De Haas and Popov (2019) offered worldwide evidence supporting that countries with bank-dominant financial systems can promote green innovation by expanding green bond issuance.

On the other side, many studies also focus directly on how green innovation mitigates carbon emissions. For example, Guo et al. (2021) employed a China’s provincial panel dataset and suggested a stable long-term inhibitory impact of green innovation on carbon emissions. Using evidence from European and African countries, previous studies have extensively confirmed that green innovation can reduce carbon emissions (Álvarez-Herránz et al. 2017; Mensah et al. 2018; Töbelmann and Wendler 2020; Obobisa et al. 2022). Similar findings are provided by Razzaq et al. (2021) using BRICS countries’ evidence. In addition, Álvarez-Herránz et al. (2017) identified green innovation as an important factor affecting the sustainable transformation of a country’s economic structure, and Balsalobre-Lorente et al. (2018) argued that green innovation is vital to alleviate environmental pollution due to increased economic activities.

From a policy point of view, it has been widely recognized that environmental regulations are an important tool for regulating environmental protection behavior, which significantly influence enterprises’ production and investment activities (Greenstone et al. 2012; Hancevic 2016). Porter (1991) argued that appropriate environmental regulations usually induce firms to engage in more innovative activities and increase their productivity, thus offsetting the costs incurred by environmental protection and increasing their profitability in the market. Maxwell and Decker (2006) discovered that enterprises would invest in environmental protection for the purpose of reducing environmental compliance costs. Caparrós et al. (2013) indicated that enterprises usually reduce production to ensure that their emissions are within a given emission limit. Consequently, environmental regulations have been gradually incorporated into carbon emission-related studies with the increasing prominence of environmental problems and the increasing governmental attention to environmental protection, especially in a large carbon emitting country like China. For example, Li and Shen (2008) supported the role of the pollution charge system in reducing pollution emissions in China. Zhao et al. (2009) viewed environmental regulations as a constraint imposed on individuals or organizations for the purpose of environmental protection, which can be classified into explicit and implicit environmental regulations. Shen et al. (2017) found that carbon emission trading policies can effectively mitigate enterprises’ carbon emissions.

Despite the above fruitful findings, a close inspection of previous studies reveals the following two research gaps that can be filled to enrich the existing literature regarding how green finance and green innovation affect carbon emissions. First, most of the extant studies are empirical but they lack a unified theoretical framework for examining how green finance, green innovation, environmental regulations, and carbon emissions interact. To fill this gap, this study theoretically constructs an endogenous growth model to discover how green finance and green innovation mitigate carbon emissions as well as the moderating mechanism of environmental regulations. Second, previous studies mainly take Chinese provincial panel datasets to conduct empirical analysis. To overcome this weakness, this study uses China’s municipal-level panel data in more detail to include the potential heterogeneity among different geographic locations in terms of resource endowments, development patterns, capital, and technology capacities.

## Endogenous growth model and research hypotheses

Referring to Bovenberg and Smulders (1995), this study incorporates the natural capital into Romer’s endogenous growth model (Romer 1990) and proposes an analytical framework that clearly illustrates the impact of green finance and green innovation on CO_2_ emissions.

### Final product production sector

The final product market is assumed to be a perfectly competitive market. Enterprises in the market use production factors (i.e., intermediate products, human capital, and natural capital) to produce a single final product. As a result, the aggregate Cobb–Douglas production function can be specified as:

1 $$Y={H}_{Y}^{\alpha }{E}^{\beta }{\int }_{0}^{A}X{(i)}^{1-\alpha -\beta }di$$

where *Y* is the total output; $H_{Y}$ and *E* denote human capital and natural capital, respectively; and $0 < \alpha ,\beta < 1$ should be satisfied. Moreover, it is assumed that natural capital (*E*) is a function of natural resources (*N*) and pollution emissions (*Z*), that is, $E = F(N,Z)$ with the conditions of $\partial F/\partial N > 0$ and $\partial F/\partial Z < 0$ (Bovenberg and Smulders 1995; Shi and Shi 2022). $X(i)$ represents the quantity of the intermediate product input *i*, and *A* is the type of intermediate product inputs.

According to Romer’s endogenous growth model, people can only produce products with existing knowledge, and production beyond existing knowledge can only be achieved through technological innovation. *A* in Eq. (1) reflects the level of technology or the stock size of knowledge. A higher *A* indicates the presence of technological innovation in the society, the greater variety of intermediate product $X(i)$, and the higher total output *Y*. Let the price of the final product be 1, and then, Eq. (2) indicates the profit function $\left( {\pi_{Y} } \right)$ of the final product production sector.

2 $$\pi_{Y} = Y - \omega_{H} - P_{E} E - \int_{0}^{A} {P({\text{i}})X(i)di}$$

where $\omega_{H}$ and $P_{E}$ denote the cost of human capital and the price of natural capital, respectively. The price of the intermediate product input *i* is $P({\text{i}})$, and its quantity is written as Eq. (3) based on the first-order necessary condition for profit maximization.

3 $$X(i) = \left[ {\frac{{(1 - \alpha - \beta )H_{Y}^{\alpha } E^{\beta } }}{P(i)}} \right]^{{\frac{1}{\alpha + \beta }}}$$

### Intermediate product production sector

Suppose that the intermediate product production sector consists of monopolistic manufacturers. Following Romer (1990) and Peng and Bao (2006), producing one unit of intermediate product requires $\varepsilon$ units of final product inputs and the use of new technology. Let the cost of applying the new technology be *k* times of the price of the final product. Then, the profit function $\left( {\pi (i)} \right)$ of the intermediate product is given as:

4 $$\pi (i) = P(i)X(i) - \varepsilon (1 + k)X(i)$$

From the first-order necessary condition for profit maximization of the intermediate product and Eq. (3), the optimal quantity and price of the intermediate product can be written as Eqs. (5) and (6), respectively.

5 $$X(i) = \left[ {\frac{{(1 - \alpha - \beta )^{2} H_{Y}^{\alpha } E^{\beta } }}{\varepsilon (1 + k)}} \right]^{{\frac{1}{\alpha + \beta }}}$$

6 $$P(i) = \frac{\varepsilon (1 + k)}{{1 - \alpha - \beta }}$$

Substituting Eqs. (5) and (6) into Eq. (4), the maximum profit of the intermediate product producer is expressed as Eq. (7).

7 $$\pi (i) = (\alpha + \beta )(1 - \alpha - \beta )^{{\frac{2 - \alpha - \beta }{{\alpha + \beta }}}} (1 + k)^{{\frac{\alpha + \beta - 1}{{\alpha + \beta }}}} \varepsilon^{{\frac{\alpha + \beta - 1}{{\alpha + \beta }}}} H_{Y}^{{^{{\frac{\alpha }{\alpha + \beta }}} }} E^{{\frac{\beta }{\alpha + \beta }}}$$

Let *T* be $(\alpha + \beta )(1 - \alpha - \beta )^{{\frac{2 - \alpha - \beta }{{\alpha + \beta }}}} (1 + k)^{{\frac{\alpha + \beta - 1}{{\alpha + \beta }}}} \varepsilon^{{\frac{\alpha + \beta - 1}{{\alpha + \beta }}}}$. Equation (7) can be simplified as:

8 $$\pi (i) = TH_{Y}^{{^{{\frac{\alpha }{\alpha + \beta }}} }} E^{{\frac{\beta }{\alpha + \beta }}}$$

Whether or not the intermediate product producer uses new technology for production depends on the return on the use of new technology for production $\left( {V_{t} } \right)$, which is equal to the present value of the intermediate product profit in Eq. (9).

9 $$V_{t} = \int_{t}^{\infty } {\pi_{i} (\tau )e^{{ - \int_{t}^{\tau } {r(s)ds} }} d\tau }$$

where $\pi_{i} (\tau )$ represents the profit of the intermediate product input *i* at time $\tau$. $r$ is the time-varying equilibrium interest rate that is discounted using continuous compounding. Taking the first-order derivatives of both sides of Eq. (9) with respect to *t* yields:

10 $$\pi (i) = \dot{V} + rV \Rightarrow r = \frac{{\pi (i) - \dot{V}}}{V}$$

where $\dot{V}$ represents the first-order derivative of *V* with respect to *t*.

### Research and development sector

As shown in Eq. (7), the profit of the intermediate product producers is directly related to natural resources and pollution emissions. That is, the intermediate product producers’ demand for technology (*A*) will be subject to the environment and resources constraints. As a result, it is assumed that the R&D sector favors green knowledge production or green technological innovation by taking the form of knowledge accumulation as below (Romer 1990).

11 $$\dot{A} = \delta H_{A} A$$

where $\delta$ and $H_{A}$ denote the R&D sector’s productivity and human capital, respectively. *A* is the existing stock of knowledge.

Since the intermediate product production sector needs to purchase knowledge from the research and development (R&D) sector, the equilibrium price of knowledge $\left( {P_{A} } \right)$ can be expressed as Eq. (12).

12 $$P_{A} (t) = V_{t} = \int_{t}^{\infty } {\pi_{i} (\tau )e^{{ - \int_{t}^{\tau } {r(s)ds} }} d\tau }$$

That is, $P_{A}$ can be obtained by discounting the intermediate product production sector’s profits. Taking the first-order derivatives of both sides of the equilibrium price of knowledge in Eq. (12) with respect to *t* and combining the definition of equilibrium growth path and Eq. (8) yields:

13 $$P_{A} = \frac{\pi (i)}{r} = \frac{T}{r}H_{{_{Y} }}^{{\frac{\alpha }{\alpha + \beta }}} E^{{\frac{\beta }{\alpha + \beta }}}$$

For the R&D sector, whether to undertake R&D innovation depends on whether the price of knowledge $\left( {P_{A} } \right)$ in Eq. (13) is optimal. From Eqs. (1) and (13), it can be seen that $P_{A}$ is related to the interest rate (*r*) and natural capital input (*E*). That is, a lower *r* results in a higher $P_{A}$, which provides more incentive for the R&D sector to undertake R&D activities. Furthermore, the greener the R&D sector’s knowledge production is, i.e., a lower amount of pollution emissions (*Z*), the higher $P_{A}$ and profit will be. This is because the natural capital input $\left( {E = F\left( {N,Z} \right)} \right)$ is a decreasing function of *Z*, i.e., $\partial F/\partial Z < 0$. That is, a higher *E* will lead to a higher $P_{A}$ and profit, which motives the R&D sector to first consider producing green knowledge or green technological innovations.

### Green finance

In general, the production project valuation of intermediate product manufacturers depends on the initial project valuation ($I_{0}$) and the existing knowledge level (*A*; technological level). As a result, an enterprise with better basic production technologies is more likely to have higher production project financing that can be assumed as:

14 $$I = I_{0} A^{\nu }$$

where *v* is positive and $I_{0}$ includes the patents, physical materials, and human capital that the enterprise needs to purchase.

It is assumed that the enterprise obtains the required production funds through external financing with the financing cost of $r_{1}$. Then, the marginal cost of external financing is given as:

15 $$\int_{t}^{\infty } {r_{1} I_{0} A^{\nu } } e^{ - r(s - t)} ds = \frac{{I_{0} A^{\nu } r_{1} }}{r}$$

where *r* is the equilibrium interest rate. As seen in Eq. (8), the profit of the intermediate product production sector is influenced by natural capital, indicating that enterprises can increase their profits by adopting green production technologies with low pollution emissions. However, green technology innovation needs significant financial support, and green finance is believed to play a vital role in providing such support. Broadly speaking, financial institutions in a region with higher green finance are more likely to provide enterprises with “green loan interest rates” below the market average rate to support their green transformation. Following Shi and Shi (2022), the relationship between the green loan interest rate $\left( {r_{1} } \right)$, equilibrium interest rate (*r*), and green finance $\left( \eta \right)$ is assumed as follows:

16 $$r_{1} = \frac{r}{\eta }$$

where $\eta$ represents the level of green finance development and $0 < \eta < 1$ should be satisfied. As shown in Eq. (16), $\partial r_{1} /\partial \eta < 0$ implies that the higher $\eta$, the lower the actual interest rate, i.e., the green loan interest rate, required for enterprises to finance their green projects. Thus, compared with a region with a low $\eta$, the enterprise’s green project financing cost in a region with a high $\eta$ is lower. As a result, Eq. (17) can be obtained by substituting Eq. (16) into Eq. (15).

17 $$\int_{t}^{\infty } {r_{1} I_{0} A^{\nu } } e^{ - r(s - t)} ds = \frac{{I_{0} A^{\nu } }}{\eta }$$

For intermediate product production enterprises, they are willing to adopt green production technologies only when the present value of corporate financing cost is not greater than the present value of corporate profit, i.e., $\frac{{I_{0} A^{\nu } }}{\eta } \le V$. Accordingly, the critical state in which enterprises are willing to adopt green production technology is:

18 $$\frac{{I_{0} A^{\nu } }}{\eta } = V$$

### Environmental regulations

Previous studies also confirm that environmental regulations influence a region’s green finance (Cooray 2011; Li 2021). We follow Han (2003) and incorporate environmental regulations (*ER*) into the green finance development model in Eq. (19).

19 $$\dot{\eta } = \gamma (ER)H_{\eta } \eta$$

where $\gamma$ is the green finance efficiency. $\partial \gamma /\partial ER > 0$ indicates that environmental regulations are conducive to improving the green finance efficiency, and $H_{\eta }$ represents the human capital of the green finance sector.

Taking the logarithm and the first-order derivatives of both sides of Eq. (18) with respect to *t* yields:

20 $$\frac{{\dot{V}}}{V} = \nu \frac{{\dot{A}}}{A} - \frac{{\dot{\eta }}}{\eta }$$

From Eqs. (10), (18) and (20), the growth rate of technical knowledge level $\left( {g_{A} } \right)$ is:

21 $$g_{A} = \frac{{\dot{A}}}{A} = \frac{{TH_{Y}^{{\frac{\alpha }{\alpha + \beta }}} E^{{\frac{\alpha }{\alpha + \beta }}} }}{{\nu I_{0} A^{\nu } }}\eta - \frac{r}{\nu } + \gamma (ER)H_{\eta }$$

### CO_2_ emissions

According to Grossman and Krueger (1995), green technological progress usually affects pollution emissions in the following two ways. First, it increases productivity and improves resource use efficiency, which in turn weakens the environmental impact of production activities. Second, it replaces environmentally unfriendly technologies and reduces pollution emissions per unit of output. Hence, the improving green technologies reduce enterprises’ carbon emissions, which is empirically supported by Wei and Yang (2010). Referring to Brock and Taylor (2005), CO_2_ emissions are assumed as:

22 $$\mathop \Omega \limits^{ \bullet } = - g_{A} \Omega \Rightarrow g_{\Omega } = - g_{A}$$

where $\Omega$ is CO_2_ emissions and $g_{\Omega }$ is the growth rate of CO_2_ emissions. Finally, the following key equations are put together for derivation and $g_{\Omega }$ can be rewritten as Eq. (23).

23 $$\begin{gathered} \left\{ \begin{gathered} \begin{array}{*{20}c} {} & {} \\ \end{array} \pi (i) = TH_{Y}^{{^{{\frac{\alpha }{\alpha + \beta }}} }} E^{{\frac{\beta }{\alpha + \beta }}} \begin{array}{*{20}c} {} \\ \end{array} \begin{array}{*{20}c} {} \\ \end{array} \begin{array}{*{20}c} {} & {} \\ \end{array} \begin{array}{*{20}c} {} \\ \end{array} \begin{array}{*{20}c} {\begin{array}{*{20}c} {} \\ \end{array} \begin{array}{*{20}c} {\begin{array}{*{20}c} {} \\ \end{array} \begin{array}{*{20}c} {} \\ \end{array} } \\ \end{array} } \\ \end{array}\hfill \\ \begin{array}{*{20}c} {} & {} \\ \end{array} \frac{{\mathop V\limits^{ \bullet } }}{V} = \frac{\pi (i)}{V} - r\begin{array}{*{20}c} {} \\ \end{array} \begin{array}{*{20}c} {} \\ \end{array} \begin{array}{*{20}c} {} \\ \end{array} \begin{array}{*{20}c} {} \\ \end{array} \begin{array}{*{20}c} {} \\ \end{array} \begin{array}{*{20}c} {} \\ \end{array} \begin{array}{*{20}c} {} \\ \end{array} \begin{array}{*{20}c} {} \\ \end{array} \begin{array}{*{20}c} {} \\ \end{array} \begin{array}{*{20}c} {} \\ \end{array} \begin{array}{*{20}c} {} \\ \end{array} \begin{array}{*{20}c} {\begin{array}{*{20}c} {} \\ \end{array} \begin{array}{*{20}c} {} \\ \end{array} } \\ \end{array}\hfill \\ \begin{array}{*{20}c} {} & {} \\ \end{array} \frac{{\mathop \eta \limits^{ \bullet } }}{\eta } = \gamma (ER)H_{\eta } \begin{array}{*{20}c} {} \\ \end{array} \begin{array}{*{20}c} {} \\ \end{array} \begin{array}{*{20}c} {} \\ \end{array} \begin{array}{*{20}c} {} \\ \end{array} \begin{array}{*{20}c} {} \\ \end{array} \begin{array}{*{20}c} {} \\ \end{array} \begin{array}{*{20}c} {\begin{array}{*{20}c} {} \\ \end{array} \begin{array}{*{20}c} {} \\ \end{array} \begin{array}{*{20}c} {} \\ \end{array} \begin{array}{*{20}c} {} \\ \end{array} } \\ \end{array} \begin{array}{*{20}c} {} \\ \end{array}\hfill \\ \begin{array}{*{20}c} {} & {} \\ \end{array} \frac{{\mathop V\limits^{ \bullet } }}{V} = \nu \frac{{\mathop A\limits^{ \bullet } }}{A} - \frac{{\mathop \eta \limits^{ \bullet } }}{\eta }\begin{array}{*{20}c} {} \\ \end{array} \begin{array}{*{20}c} {} \\ \end{array} \begin{array}{*{20}c} {} \\ \end{array} \begin{array}{*{20}c} {} \\ \end{array} \begin{array}{*{20}c} {} \\ \end{array} \begin{array}{*{20}c} {} \\ \end{array} \begin{array}{*{20}c} {} \\ \end{array} \begin{array}{*{20}c} {} \\ \end{array} \begin{array}{*{20}c} {} \\ \end{array} \begin{array}{*{20}c} {} \\ \end{array} \begin{array}{*{20}c} {} \\ \end{array} \begin{array}{*{20}c} {\begin{array}{*{20}c} {\begin{array}{*{20}c} {} \\ \end{array} } \\ \end{array} } \\ \end{array}\hfill \\ \begin{array}{*{20}c} {} & {} \\ \end{array} g_{A} = \frac{{\mathop A\limits^{ \bullet } }}{A}\begin{array}{*{20}c} {} \\ \end{array} \begin{array}{*{20}c} {} \\ \end{array} \begin{array}{*{20}c} {} \\ \end{array} \begin{array}{*{20}c} {} & {} \\ \end{array} \begin{array}{*{20}c} {} \\ \end{array} \begin{array}{*{20}c} {} \\ \end{array} \begin{array}{*{20}c} {} \\ \end{array} \begin{array}{*{20}c} {} \\ \end{array} \begin{array}{*{20}c} {} \\ \end{array} \begin{array}{*{20}c} {} \\ \end{array} \begin{array}{*{20}c} {} \\ \end{array} \begin{array}{*{20}c} {} \\ \end{array} \begin{array}{*{20}c} {} \\ \end{array}\hfill \\ \begin{array}{*{20}c} {} & {} \\ \end{array} g_{\Omega } = - g_{A} \begin{array}{*{20}c} {} & {} \\ \end{array} \begin{array}{*{20}c} {} & {} \\ \end{array} \begin{array}{*{20}c} {} & {} \\ \end{array} \begin{array}{*{20}c} {} & {} \\ \end{array}\hfill \\ \end{gathered} \right. \hfill \\ g_{\Omega } = - g_{A} = - \frac{{TH_{Y}^{{\frac{\alpha }{\alpha + \beta }}} E^{{\frac{\alpha }{\alpha + \beta }}} }}{{\nu I_{0} A^{\nu } }}\eta + \frac{r}{\nu } - \gamma (ER)H_{\eta } \hfill \\ \end{gathered}$$

From Eq. (23), green finance reduces CO_2_ emissions, and technological innovation ($A$) is also an important factor of carbon emission reduction. Taking the first-order partial derivatives of Eq. (23) with respect to $ER$ yields:

24 $$\frac{{\partial g_{\Omega } }}{\partial ER} = \frac{{\partial g_{\Omega } }}{\partial \gamma }\frac{\partial \gamma }{{\partial ER}} = - H_{\eta } \frac{\partial \gamma }{{\partial ER}} < 0$$

From Eqs. (19) and (24), environmental regulations reduce CO_2_ emissions by enhancing green finance efficiency. Then, the following two research hypotheses can be proposed based on the results and analysis of the model derived above.

- **Hypothesis 1**: Both green finance and green innovation can inhibit CO_2_ emissions.
- **Hypothesis 2**: Environmental regulations can strengthen the inhibitory impact of green finance on CO_2_ emissions.

According to Li and Ye (2019), the rapid development of green finance in China depends largely on the government’s institutional change, which is a kind of “top-down” model. In this sense, exploring how environmental regulations moderate the inhibitory impact of green finance on CO_2_ emissions holds great practical significance. Nevertheless, unlike the development model of green finance, it has been widely observed that green innovation is mainly driven by listed companies’ green patents in China, which is, to a larger extent, a kind of market behavior. As a result, research attention has gradually shifted from governmental behavior to market behavior in order to explore the potential mechanisms through which the inhibitory impact of green innovation on CO_2_ emissions is moderated. The existing literature has identified the regional marketization level as a moderating variable. For example, Zeng et al. (2021) found that marketization levels positively promote green innovation in China. Therefore, the following research hypothesis can be proposed.

- **Hypothesis 3**: Marketization levels can strengthen the inhibitory impact of green innovation on CO_2_ emissions.

## Methodology

### Empirical model specification

The following benchmark model is proposed to empirically examine how green finance and green innovation affect CO_2_ emission intensity to test Hypothesis 1.

25 $$CO_{2it} = a_{0} + a_{1} GF_{it} + a_{2} GI_{it} + {\mathbf{Control}}_{it} {{\varvec{\upbeta}}} + u_{i} + \nu_{t} + \varepsilon_{it}$$

where $CO_{2it}$ is CO_2_ emission intensity. $GF_{it}$, $GI_{it}$, and ${\mathbf{Control}}_{it}$ represent green finance, green innovation, and the row vector of control variables, respectively. Equation (25) considers fixed effects for individual $\left( {u_{i} } \right)$ and time $\left( {\nu_{t} } \right)$. $\varepsilon_{it}$ indicates the random error term.

To further test Hypothesis 2, Eq. (26) is proposed by incorporating environmental regulations $\left( {ER_{it} } \right)$ and its interaction term with green finance $\left( {GF_{it} \times ER_{it} } \right)$ into Eq. (25). Similarly, Eq. (27) is proposed by incorporating marketization levels $\left( {Market_{it} } \right)$ and its interaction term with green innovation $\left( {GI_{it} \times Market_{it} } \right)$ into Eq. (25) to test Hypothesis 3. Other variables are the same as above.

26 $$CO_{2it} = a_{0} + a_{1} GF_{it} + a_{2} GI_{it} + a_{3} ER_{it} + a_{4} GF_{it} \times ER_{it} + {\mathbf{Control}}_{it} {{\varvec{\upbeta}}} + u_{i} + \nu_{t} + \varepsilon_{it}$$

27 $$CO_{2it} = a_{0} + a_{1} GF_{it} + a_{2} GI_{it} + a_{3} Market_{it} + a_{4} GI_{it} \times Market_{it} + {\mathbf{Control}}_{it} {{\varvec{\upbeta}}} + u_{i} + \nu_{t} + \varepsilon_{it}$$

### Variables and data sources

#### Dependent variable: CO_2_ emission intensity

Referring to Han and Xie (2017), the dependent variable is CO_2_ emission intensity, which is measured by the logarithm of each city’s carbon emissions. Equation (28) shows its calculation using the consumption of electricity $\left( {C_{e} } \right)$, natural gas $\left( {C_{n} } \right)$, and liquefied petroleum gas $\left( {C_{p} } \right)$ with amounts of consumption $E_{e}$, $E_{n}$, and $E_{p}$, respectively.

28 $$I = C_{n} + C_{p} + C_{e} = kE_{n} + \gamma E_{p} + \varphi (\eta \times E_{e} )$$

where *I* is carbon emissions. *k* and $\gamma$ are the emission factors of natural gas (2.1622 kg CO_2_/m^3^) and liquefied petroleum gas (3.1013 kg CO_2_/m^3^). $\varphi$ is the coal power fuel chain’s greenhouse gas emission factor, which is equivalent to 1.3203 kg/(kW h) CO_2_ emissions. The China Electric Power Yearbook provides the proportion of coal power generation $\left( \eta \right)$.

#### Core independent variables: green finance and green innovation

As one of the core independent variables, green finance (*GF*) can be collected by performing the following steps (Wen et al. 2021): (1) Based on the China Stock Market & Accounting Research (CSMAR) database, we extracted keywords (e.g., environmental protection, recycling, photovoltaics, and environmental engineering) from the Statistical Classification of Energy Conservation, Environmental Protection and Cleaning Industries (2021); (2) these keywords were matched with the main business information of Chinese A-share-listed companies, yielding 675 environmental protection enterprises; and (3) the screened listed companies were grouped into prefecture-level cities according to the industrial and commercial registration place. Meanwhile, the ratio of the total amount of liabilities of environmental protection enterprises in the city to the total amount of liabilities of the listed companies in the city was employed as a proxy for each city’s green finance.

The other core independent variable is green innovation (*GI*) in this study, which can be obtained by taking the following steps: (1) We obtained the data on all Chinese-listed companies’ patent applications, granted patents, and patent International Patent Classification (IPC) codes from the China Center for Economic Research (CCER) database; (2) using the green patent IPC codes in the IPC Green Inventory released by the World Intellectual Property Organization (WIPO) in 2010, the listed companies’ patent IPC codes were matched to obtain the data on each company’s patent applications and granted green patents in each year; (3) similar to green finance index construction, the total number of the city’s listed companies’ green patents represents green innovation in the prefecture-level city.

#### Moderating and control variables

Following Xie (2021), this study employs the proportion of industrial pollution control investment in regional industrial GDP as a measure of environmental regulations (*ER*) to conduct a moderating mechanism analysis. In addition, to better capture how green finance and green innovation influence carbon emissions, this study refers to the existing literature and considers a range of control variables such as the local public finance expenditure (*FE*), regional GDP (*GDP*), industrial structure (*STR*) measured as the ratio of value added in the secondary industry to GDP, the number of employees in the secondary industry (Sec*Pop*), local education spending (*Edu*), and the number of industrial enterprises (*IE*) (Yan et al. 2016; Jiang et al. 2020; Liu et al. 2020; Guo et al. 2021; You et al. 2022).

Considering the availability, completeness, and comparability of data, this study uses a panel dataset consisting of 107 Chinese prefecture-level cities over 2010 − 2019. The main data sources include China Statistical Yearbook, China City Statistical Yearbook, China Energy Statistical Yearbook, and provinces and cities’ statistical bulletins. For the missing data, an interpolation approach is applied to complete the data. Table 1 shows a summary of all variables in terms of mean, medium and variance.

**Table 1:** Descriptive statistics of variables

Variable	Observation	Mean	Medium	Variance
*CO*_2_	1070	3.58	3.62	0.70
*GF*	1070	0.25	0.13	0.27
*GI*	1070	27.03	5.00	77.59
*GDP*	1070	17.25	17.21	0.86
*FE*	1070	14.73	14.69	1.04
*Edu*	1070	13.51	13.47	0.79
Sec*Pop*	1070	12.73	12.72	0.92
*IE*	1070	7.34	7.38	0.97

## Empirical results and discussions

### Results of the benchmark model

The benchmark model in Eq. (25) is estimated, and Table 2 reports the estimation results. According to the full-sample estimation in column (1), the significant and negative coefficients of *GF* and *GI* show that green finance and green innovation significantly decrease CO_2_ emissions in China’s prefecture-level cities. To investigate how green finance and green innovation influence CO_2_ emissions heterogeneously, the median of green finance (*GF*) is employed to divide the full sample into two subsamples of regions with high/low green finance (i.e., the city’s *GF* is greater/less than the median). Columns (2) and (3) of Table 2 present their respective estimation results. It can be seen that *GF* is negatively significant in column (2) and positively insignificant in column (3), while *GI* is negatively insignificant in column (2) and negatively significant in column (3), confirming the above heterogeneity. The possible reason is as follows: Enterprises in a region with a low level of green finance development, Zhang et al. (2022a, b) found that the usually faced serious financing difficulties and enterprises’ innovation advantages are more likely to be enlarged, leading to the main position of green innovation in reducing carbon emissions. As a result, enterprises can undertake various innovative activities to reduce emissions. With the ever-increasing level of green finance, enterprises’ financing pressure has been eased and the carbon emission reduction effect of green finance gradually increases. Then, more and more enterprises can use the funds raised by green finance to undertake energy saving and emission reduction projects, so that green finance becomes the main force for reducing CO_2_ emissions.

**Table 2:** Results of the benchmark model

	(1)	(2)	(3)
Variables	Full sample	High level of green finance	Low level of green finance
*GF*	− 0.0823**	− 0.0814**	0.0110
	(0.029)	(0.031)	(0.066)
*GI*	− 0.0003***	− 0.0001	− 0.0004***
	(0.000)	(0.000)	(0.000)
Sec*Pop*	0.0118	− 0.0171	0.0547***
	(0.010)	(0.013)	(0.016)
*STR*	− 0.0012	− 0.0032**	0.0002
	(0.001)	(0.001)	(0.001)
*IE*	− 0.0434**	− 0.0457	− 0.0429*
	(0.019)	(0.030)	(0.025)
*GDP*	0.0993**	0.2067***	0.0213
	(0.031)	(0.046)	(0.044)
*FE*	− 0.0601**	− 0.0245	− 0.0760**
	(0.022)	(0.030)	(0.031)
*Edu*	0.1043***	0.0113	0.1847***
	(0.029)	(0.039)	(0.040)
*cons*	1.5667***	0.9887	1.4251**
	(0.441)	(0.696)	(0.563)
Time effect	yes	yes	yes
Individual fixed effect	yes	yes	yes
*N*	1070	400	670

Standard errors are presented in parentheses. *p < 0.1, **p < 0.05, and ***p < 0.001

Considering that China is a vast country with different human, economic, and geographical characteristics in different regions, green finance and green innovation affect CO_2_ emissions differently across regions. To validate this, eastern, central, and western groups are formed and columns (1)–(3) of Table 3 report their respective estimation results. As observed, *GF* and *GI* are significantly negative in columns (1) and (2), implying that the promotion of green finance and green innovation reduces carbon emissions in eastern and central cities, but their mitigating effects are insignificant in western cities due to insignificant *GF* and *GI* in column (3). The possible reasons for the above findings are as follows: Compared with China’s western cities, it is more likely to see a sound green finance system in eastern and central cities, thereby offering more convenient financial services for enterprises to perform green production activities. Meanwhile, eastern and central cities are usually more modernized with abundant human resources. These advantages promote the tertiary industries, reduce energy consumption, and upgrade industrial structure, thereby bringing significant carbon emission reduction in eastern and central cities. Additionally, most of the eastern and central cities have the strength and manufacturing capacity of high-tech R&D, which lays a solid foundation for enterprises’ green technology innovation in these cities. In contrast, westerns cities’ backward technology environment makes it difficult for enterprises to carry out green innovation, thereby bringing challenges to carbon emission reduction through green technology innovation in these cities. The above discussions empirically confirm Hypothesis 1: Both green finance and green innovation can inhibit CO_2_ emissions.

**Table 3:** Results of the benchmark model by regions

	(1)	(2)	(3)
Variable	Eastern cities	Central cities	Western cities
*GF*	− 0.1513**	− 0.1125***	− 0.0722
	(0.059)	(0.036)	(0.066)
*GI*	− 0.0003***	− 0.0006**	0.0007
	(0.000)	(0.000)	(0.001)
Sec*Pop*	0.0075	− 0.0080	0.0271
	(0.015)	(0.022)	(0.018)
*STR*	0.0001	− 0.0038***	− 0.0030**
	(0.001)	(0.001)	(0.001)
*IE*	− 0.0601**	0.0346	− 0.0575*
	(0.031)	(0.036)	(0.032)
*GDP*	0.0195	0.2647***	0.0853*
	(0.055)	(0.057)	(0.047)
*FE*	− 0.0424	− 0.1333***	0.0565
	(0.037)	(0.033)	(0.036)
*Edu*	0.1757***	0.1180**	0.0102
	(0.049)	(0.046)	(0.044)
*cons*	1.9677***	− 0.7094	1.3230**
	(0.718)	(0.963)	(0.618)
Time effect	yes	yes	yes
Individual fixed effect	yes	yes	yes
*N*	580	270	220

Standard errors are presented in parentheses. *p < 0.1, **p < 0.05, and ***p < 0.001

### Moderating mechanism analysis of environmental regulations

After estimating the empirical model in Eq. (26), Table 4 presents the estimation results of the moderating mechanism analysis of environmental regulations for the full sample, eastern, central, and western cities in columns (1)–(4), respectively. It can be seen from column (1) that significantly negative coefficients of *GF* and $GF\times ER$ indicate that environmental regulations are a moderating mechanism through which green finance inhibits CO_2_ emissions. That is, green finance mitigates CO_2_ emissions greater in a city with stronger environmental regulations. For a 1% increase in environmental regulations, the above mitigating effect will increase by 1.52%, which empirically confirms the Hypothesis 2: Environmental regulations can strengthen the inhibitory impact of green finance on CO_2_ emissions.

**Table 4:** Results of the moderating mechanism analysis of environmental regulations

	(1)	(2)	(3)	(4)
Variable	Full sample	Eastern cities	Central cities	Western cities
*GF*	− 0.1837^***^	− 0.2547^**^	− 0.2047^**^	− 0.1023
	(0.047)	(0.105)	(0.066)	(0.105)
*GI*	− 0.0004***	− 0.0004***	− 0.0007**	0.0007
	(0.000)	(0.000)	(0.000)	(0.001)
*ER*	0.0050**	0.0066**	0.0032	0.0017
	(0.002)	(0.003)	(0.005)	(0.005)
$GF\times ER$	− 0.0152***	− 0.0159	− 0.0146*	− 0.0044
	(0.006)	(0.013)	(0.008)	(0.012)
Sec*Pop*	0.0121	0.0068	− 0.0088	0.0273
	(0.010)	(0.015)	(0.022)	(0.018)
*STR*	− 0.0012	0.0002	− 0.0038***	− 0.0029**
	(0.001)	(0.001)	(0.001)	(0.001)
*IE*	− 0.0386**	− 0.0540*	0.0368	− 0.0578*
	(0.019)	(0.031)	(0.036)	(0.033)
*GDP*	0.0969***	0.0206	0.2551***	0.0849*
	(0.031)	(0.055)	(0.057)	(0.048)
*FE*	− 0.0602***	− 0.0428	− 0.1388***	0.0588
	(0.022)	(0.037)	(0.033)	(0.037)
*Edu*	0.1070***	0.1772***	0.1433***	0.0070
	(0.028)	(0.048)	(0.048)	(0.045)
*cons*	1.5670***	1.9353***	− 0.7814	1.3474**
	(0.440)	(0.716)	(0.959)	(0.635)
Time effect	Yes	Yes	Yes	Yes
Individual fixed effect	Yes	Yes	Yes	Yes
*N*	1070	580	270	220

Standard errors are presented in parentheses. *p < 0.1, **p < 0.05, and ***p < 0.001

According to columns (2)–(4) of Table 4, the moderating effect of environmental regulations is significant only in central cities. The possible reasons are as follows: Considering that most of the central cities are in the stage of rapid development with relatively abundant financial resources, enterprises in these cities usually demonstrate strong environmental awareness and willingness to innovate. Enhancing environmental regulations can guide more green capital flows into enterprises, promote the level of green innovation, and strengthen its moderating effect. In contrast, the marginal moderating effect of environmental regulations is limited in eastern cities due to their higher existing level of green innovation and environmental awareness. Western cities usually have lower green innovation capacities and higher green innovation costs, resulting in insignificant moderating effect of environmental regulations due to relatively scarce financial resources in these cities.

To further validate the above findings, this study evaluates the policy of “China’s low-carbon city pilot program” and explores its impact on green finance. Up to now, there are three batches of low-carbon pilot cities, and the first batch was announced in 2010. However, pilot cities covered in the first batch were not considered in this study because the sample period is 2010–2019. Next, according to the National Development and Reform Commission’s guidance in the documents “Notice on the pilot work for the second batch of low-carbon provinces and low-carbon cities” and “Notice on the third batch of national low-carbon cities pilot work,” pilot cities covered in the second and third batches form the treatment group, and the other cities excluded in these two batches form the control group. The document issuance years 2013 and 2017 are used as cutoff points to investigate how policy implementation affects green finance by employing the difference-in-difference (DID) model in Eq. (29).

29 $$GF_{it} = \alpha_{0} + \alpha_{1} T{\text{ime}}_{it} \times T{\text{reat}}_{i} + {\mathbf{Control}}_{it} {{\varvec{\upbeta}}} + \lambda_{i} + \gamma_{t} + \mu_{it}$$

where the group dummy variable $T{\text{reat}}_{i}$ is defined as 1 for the treatment group and 0 for the control group and the time dummy variable $T{\text{ime}}_{it}$ is defined as 1 and 0 for the year after and before policy implementation, respectively. $\alpha_{1}$ represents the impact of China’s low-carbon city pilot program on green finance.

**Fig. 1:**
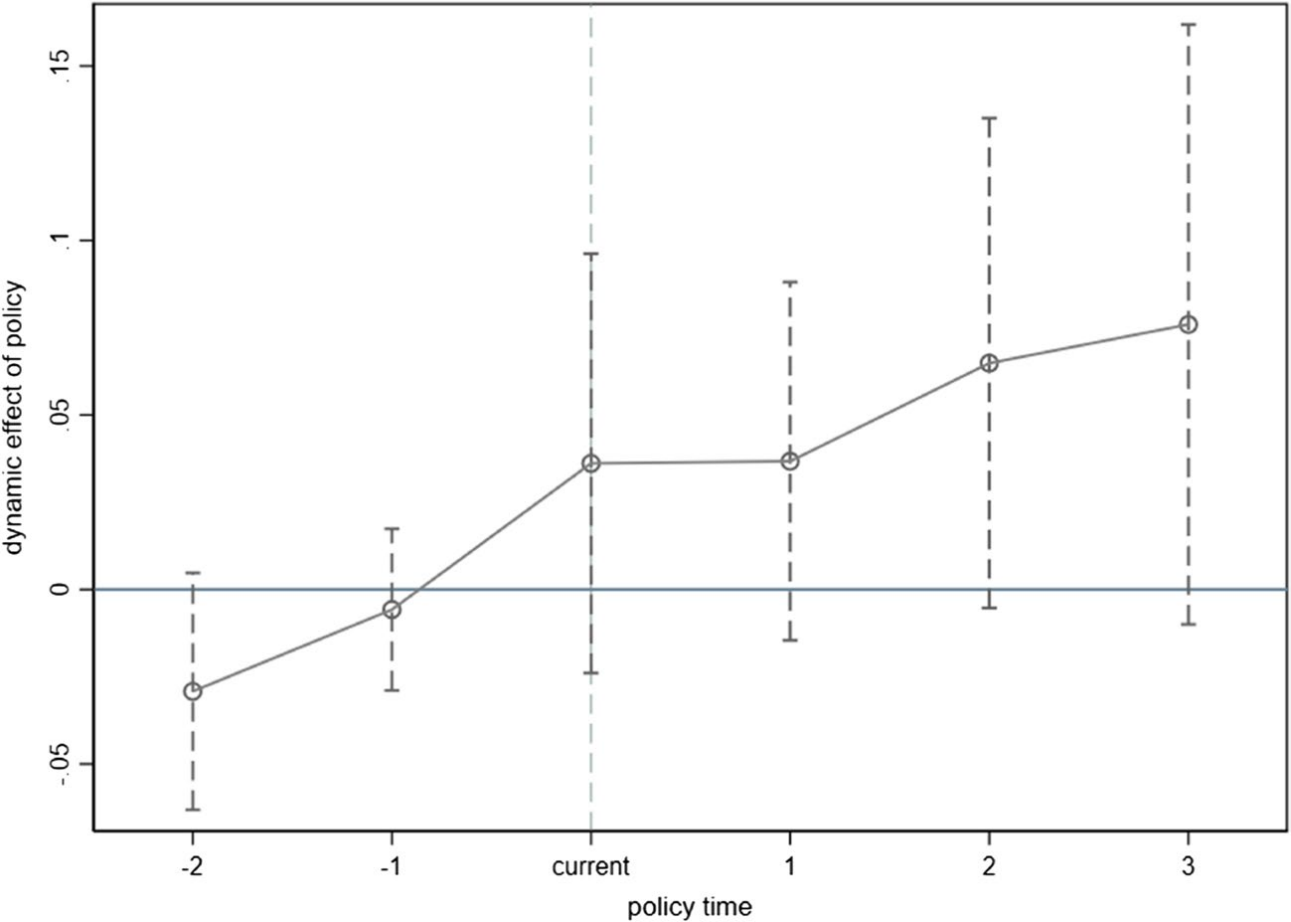
Visual inspection of the parallel trend test

The DID model in Eq. (29) also requires the treatment and control groups to meet the parallel trend assumption. In addition, a DID model with multiple time periods needs to consider different policy implementation times in different cities. To achieve this, the event study approach (ESA) is adopted to perform the parallel trend test for the DID model in Eq. (29), and Fig. 1 shows the visual inspection result over different time points. As observed, the coefficient of the interaction term is not significantly different from 0 before policy implementation because the 95% confidence interval contains the value of 0 in times − 1 and − 2. That is, there is no significant difference between the treatment and control groups before implementing the policy, which indicates that the parallel trend assumption holds. Meanwhile, a significant positive impact of policy implementation on green finance in the current period implies that the innovation-promoting effect of policy implementation has strong immediacy.

Table 5 reports the estimation result of Eq. (29). It can be seen that the coefficient of $T{\text{ime}}_{it} \times T{\text{reat}}_{i}$ is significantly positive at the 10% level, indicating that the implementation of China’s low-carbon city pilot program significantly increases the level of green finance in the pilot cities. This finding with respect to the impact of environmental regulations and green finance is consistent with previous studies. For example, Zheng (2021) found that green financial policies can help cities transform from the extensive financial development model to a green model. Zhao et al. (2023) also supported that the establishment of green financial reform and innovation pilot zones can enhance the efficiency of green finance in the pilot zones. A potential reason is that the above policy implementation reveals to some extent that green and low-carbon transitions will be the government’s future focus. This strengthens the low-carbon oriented support from the government’s fiscal policy and financial institutions’ funds, which in turn promotes the level of green finance in the region.

**Table 5:** Results of the evaluation of China’s low-carbon city pilot program

Variable	*GF*
$T{\text{ime}} \times T{\text{reat}}$	0.0203*
	(0.032)
Sec*Pop*	0.0201
	(0.052)
*STR*	− 0.0001
	(0.001)
*IE*	− 0.0372
	(0.036)
*GDP*	− 0.0461
	(0.055)
*FE*	− 0.0687***
	(0.021)
*Edu*	0.0721**
	(0.026)
*cons*	1.2143***
	(0.623)
Time effect	yes
Individual fixed effect	yes
*N*	1070

Standard errors are presented in parentheses. *p < 0.1, **p < 0.05, and ***p < 0.001

### Moderating mechanism analysis of marketization levels

Referring to Fan et al. (2003), this study constructs the indicator of marketization levels by employing the principal component analysis technique to determine weights of five scores related to government-market relationship, non-state economic development, product market development, factor market development, and intermediary organization development and laws. It is worth noting that China’s provincial marketization levels are used in this study to represent its marketization levels of prefecture-level cities due to data availability.

After decentralizing the relevant variables, Eq. (27) is estimated, and Table 6 reports the estimation results. As shown in column (1), the significantly negative coefficient of $GI\times {\text{Market}}$ indicates that marketization levels are a moderating mechanism through which green innovation inhibits CO_2_ emissions. In other words, green innovation demonstrates greater mitigating effects on CO_2_ emissions in cities with higher marketization levels. For a 1% increase in marketization levels, its mitigating effect will increase by 0.91%, which empirically confirms the Hypothesis 3: Marketization levels can strengthen the inhibitory impact of green innovation on CO_2_ emissions.

**Table 6:** Results of the moderating mechanism analysis of marketization levels

	(1)	(2)	(3)	(4)
Variable	Full sample	Eastern cities	Central cities	Western cities
*GF*	− 0.0786***	− 0.1458**	− 0.1207***	− 0.0588
	(0.029)	(0.059)	(0.035)	(0.068)
*GI*	− 0.0002	− 0.0001	− 0.0007*	− 0.0002
	(0.000)	(0.000)	(0.000)	(0.001)
$GI\times {\text{Market}}$	− 0.0091**	− 0.0104**	0.0035	0.0439
	(0.004)	(0.005)	(0.021)	(0.029)
Market	− 0.0480*	− 0.0530	− 0.1970***	0.0325
	(0.028)	(0.048)	(0.053)	(0.041)
*STR*	− 0.0013	0.0000	− 0.0044***	− 0.0027*
	(0.001)	(0.001)	(0.001)	(0.001)
*FE*	− 0.0624***	− 0.0504	− 0.1343***	0.0540
	(0.022)	(0.038)	(0.032)	(0.036)
Sec*Pop*	0.0149	0.0115	0.0016	0.0238
	(0.010)	(0.015)	(0.021)	(0.018)
*IE*	− 0.0454**	− 0.0628**	0.0379	− 0.0521
	(0.019)	(0.031)	(0.035)	(0.033)
*GDP*	0.1044***	0.0277	0.2590***	0.0829*
	(0.031)	(0.055)	(0.056)	(0.048)
*Edu*	0.1086***	0.1897***	0.1216***	0.0181
	(0.029)	(0.049)	(0.046)	(0.044)
*cons*	1.8861***	2.2257**	1.0685	0.9555
	(0.509)	(0.876)	(1.057)	(0.687)
Time effect	Yes	Yes	Yes	Yes
Individual fixed effect	Yes	Yes	Yes	Yes
*N*	1070	580	270	220

Standard errors are presented in parentheses. *p < 0.1, **p < 0.05, and ***p < 0.001

Interestingly, columns (2)–(4) of Table 6 show that the moderating effect of marketization levels is significant only in eastern cities (− 0.0104). A possible explanation is as follows: Compared to central and western cities in China, its eastern cities usually have higher marketization levels, resulting in higher resource allocation efficiency (Sun 2007; Zhang and Cui 2021). As a result, enterprises in eastern cities have more efficient access to market resources to promote green innovation activities, which reduces carbon emissions.

### Results of the robustness and endogeneity checks

To eliminate the influence of extreme values in the dataset, this study trims all independent variables at the 1% and 99% quantiles and column (1) of Table 7 reports the re-estimation results of the benchmark model in Eq. (25). As seen, the coefficients of *GF* and *GI* in column (1) are significantly negative, reflecting that green finance and green innovation still significantly reduce carbon emissions. That is, the mitigating effects of green finance and green innovation on CO_2_ emissions are robust. Alternatively, this study performs the robustness check by avoiding the influence of omitted variables. To achieve this, additional independent variables that were occasionally used in previous studies are incorporated into the benchmark model in Eq. (25) to control wealth and demographic factors, including savings $\left( {Sav} \right)$, the total population $\left( {Pop} \right)$, and the total wages $\left( {Wage} \right)$. According to column (2) of Table 7, significantly negative coefficients of *GF* and *GI* demonstrate that green finance and green innovation can still inhibit CO_2_ emissions after including these additional control variables. That is, the proposed benchmark model is robust.

**Table 7:** Results of the robustness and endogeneity checks

Variable	(1)	(2)	(3)
*GF*	− 0.0823***	− 0.0791***	
	(0.029)	(0.029)	
*GI*	− 0.0003***	− 0.0003***	
	(0.000)	(0.000)	
*LGF*			− 0.0676**
			(0.029)
*LGI*			− 0.0004***
			(0.000)
Sec*Pop*	0.0118	0.0100	0.0105
	(0.010)	(0.011)	(0.011)
*STR*	− 0.0012	− 0.0014*	− 0.0011
	(0.001)	(0.001)	(0.001)
*IE*	− 0.0434**	− 0.0476**	− 0.0626***
	(0.019)	(0.019)	(0.020)
*GDP*	0.0993***	0.1064***	0.0861***
	(0.031)	(0.031)	(0.031)
*FE*	− 0.0601***	− 0.0586***	− 0.0421*
	(0.022)	(0.022)	(0.022)
*Edu*	0.1043***	0.1005***	0.0755***
	(0.029)	(0.029)	(0.028)
*Sav*		− 0.0000***	
		(0.000)	
*Wage*		− 0.0000	
		(0.000)	
*Pop*		0.0002	
		(0.000)	
*cons*	1.5667***	1.4462***	2.1215***
	(0.441)	(0.441)	(0.449)
Time effect	Yes	Yes	Yes
Individual fixed effect	Yes	Yes	Yes
*N*	1070	1070	963

Standard errors are presented in parentheses. *p < 0.1, **p < 0.05, and ***p < 0.001

A further issue is to examine the problem of endogeneity due to reverse causality. That is, promoting green finance and green innovation can reduce carbon emissions, while cities with lower carbon emissions usually have stronger environmental awareness and they usually pay more attention to green finance and green innovation. To eliminate the potential endogeneity, this study employs one-period lagged green finance $\left( {L.GF} \right)$ and one-period lagged green innovation $\left( {L.GI} \right)$ as additional independent variables and re-estimate Eq. (25) with results in column (3) of Table 7. Significantly negative coefficients of $L.GF$ and $L.GI$ confirm that green finance and green innovation drive their inhibitory effects on carbon emissions. These findings are consistent with the benchmark results, indicating the elimination of endogeneity and reliability of findings regarding how green finance and green innovation reduce carbon emissions.

## Conclusions

To cope with the ever-increasing challenge of carbon emission reduction, this study constructs a theoretical endogenous growth model to capture how green finance and green innovation reduce CO_2_ emissions and the moderating mechanisms of environmental regulations and marketization levels and proposes three research hypotheses. The theoretical analysis and hypotheses are further empirically examined using China’s municipal-level panel data during 2010–2019. Our main results are presented below: First, both green finance and green innovation demonstrate significant inhibitory effects on CO_2_ emissions in China’s prefecture-level cities. Second, for cities with high green finance, it is observed that green finance significantly drives carbon emission reduction, while green innovation exhibits insignificant impact. For cities with low green finance, CO_2_ emissions are significantly reduced by green innovation. Nevertheless, green finance demonstrates an insignificant effect. Third, compared with western cities, improving green finance and green innovation in eastern and central cities can significantly reduce CO_2_ emissions with a diminishing marginal effect. Finally, strong environmental regulations and higher marketization levels can strengthen the role of green finance and green innovation in driving carbon emission reduction, respectively, which demonstrate clear regional heterogeneity.

The above findings based on China’s evidence provide different stakeholders both in China and in other countries with profound implications for using green finance and green innovation as an efficient approach for reducing carbon emissions. First, local governments both in China and in other countries should vigorously guide funds to green finance, increase green inputs, and promote decarbonization through green finance. For cities with various development stages of green finance, local governments should adapt to local conditions and guide enterprises to give full play to the synergy of green finance and green innovation and accelerate the progress of green transformation. Second, banks and other financial institutions both in China and in other countries should lean toward the green economy, especially increase support for environmentally friendly enterprises and energy-saving enterprises, promote green technology innovation through green financial support, make better use of the financial market, and promote enterprises’ green transformation. Third, policymakers both in China and in other countries should formulate scientific and effective environmental regulations and policies to better play environmental regulations’ moderating function. More specifically, local governments should formulate scientific and reasonable environmental protection policies; implement financial subsidies, taxes, and other economic incentives; stimulate enterprises’ enthusiasm for innovating green technologies; and improve the effectiveness of green finance policies. They should coordinate different environmental regulatory tools, better use social supervision mechanisms, and diversify environmental regulatory systems. Notably, considering that the current market systems are imperfect in most countries, they need to gradually promote market-based environmental regulations to increase public awareness of green development and enhance green technology innovation. On the one hand, developing countries (e.g., China) need to proactively learn from the excellent practices of developed countries such as the establishment of policy-oriented green financial institutions in Germany to improve their green financial systems. Meanwhile, developing countries need to optimize their laws and regulations on protecting green innovation patents, strengthen their environmental regulatory systems, and realize the green transformation of industries. On the other hand, developed countries (e.g., the USA) need to enhance green finance communication and cooperation with developing countries through proposing green finance metrics internationally, innovating green financial systems, improving the laws and regulations on green innovation patents, and strengthening their environmental regulatory systems. In addition, raising public awareness of green development and enhancing public concepts of green innovation is a key step that should be taken by all countries to tackle the environmental issues.

Despite these valuable policy implications, there are still some different ways to extend the current study. First, future efforts can be made to gradually release some assumptions of the theoretical mechanism model, making it more powerful to interpret the complex reality. The second direction aims to improve green finance indicators in Chinese prefecture-level cities when the data availability improves. Third, the future study can take a spatial econometrics perspective to empirically examine how green finance spatially affects carbon emissions.

## Data Availability

Data are available on request.
